# Neogosseidae (Gastrotricha, Chaetonotida) from the iSimangaliso Wetland Park, KwaZulu-Natal, South Africa

**DOI:** 10.3897/zookeys.315.5593

**Published:** 2013-07-10

**Authors:** M. Antonio Todaro, Renzo Perissinotto, Sarah J. Bownes

**Affiliations:** 1 Department of Life Sciences, University of Modena and Reggio Emilia, via Campi, 231/D, I-41125 Modena, Italy; 2 School of Life Sciences, University of KwaZulu-Natal, Westville Campus, Private Bag X54001, Durban 4000, South Africa; 3 DST/NRF South African Research Chair in Shallow Water Ecosystems, Nelson Mandela Metropolitan University, P.O. Box 77000, Port Elizabeth 6031, South Africa

**Keywords:** Gastrotricha, meiofauna, new species, taxonomy, *Kijanebalola*, *Neogossea*, diagnoses

## Abstract

Among the mostly benthic gastrotrichs, the Neogosseidae (Gastrotricha, Chaetonotida) are particularly interesting from an evolutionary point of view in virtue of their planktonic lifestyle; yet, they are poorly known and uncertainties concerning morphological traits hamper accurate in-group systematics. During a recent survey of meiofauna in the iSimangaliso Wetland Park, South Africa, two species of Neogosseidae were found in a freshwater pond near Charter’s Creek on the Western Shores of Lake St Lucia. Based on morphological traits, one species has been identified as *Neogossea acanthocolla*, originally described from Brazil, while the other, affiliated to the genus *Kijanebalola*, is proposed as new to science. Using a combination of differential interference contrast and scanning electron microscopy, fine anatomical details were observed and are here discussed in a larger taxonomic framework, especially regarding *Kijanebalola devestiva* **sp. n.** Results have also provided reasons for a revision of the diagnostic traits of *Kijanebalola*, *Neogossea* and the whole Family Neogosseidae. Besides expanding awareness about the biodiversity hosted by South Africa’s first UNESCO World Heritage Site, our study will be beneficial to future phylogenetic studies of the Gastrotricha based on morphology, by allowing the selection and/or a more precise character coding of traits of phylogenetic relevance.

## Introduction

Most freshwater gastrotrichs (Chaetonotida, Paucitubulatina) have an epibenthic, periphytic or interstitial lifestyle, however, there are several species that conduct a pelagic and/or semi-planktonic existence (e.g., Balsamo and Todaro 2002, Todaro and Hummon 2008). Common features of the latter species are the absence of the furcal adhesive tubes and a re-arrangement of the locomotory ciliation, which in these forms includes discrete tufts along the trunk region and at least a band of long, strong propelling cilia that encircles more or less completely the head (e.g., Kisielewski 1991). Based on additional traits, planktonic gastrotrichs are placed into three main taxa: 1) the monotypic genus *Undula* Kisielewski, 1991, provisionally considered as a distinct subfamily of the Chaetonotidae Gosse, 1864 (see Kisielewski 1991); 2) the Family Dasydytidae Daday, 1905, which currently includes seven genera and 25 species (Todaro and Tongiorgi 2013), with most characterized by long and movable spines (e.g., Kieneke and Ostmann 2012); and 3) the Family Neogosseidae Remane, 1927, which includes two genera and eight (9?) species, all bearing a pair of peculiar, club-like cephalic tentacles.

The origin and evolution of planktonic gastrotrichs remain largely unknown (Hochberg and Litavaitis 2000, Kieneke et al. 2008b); however, the long-held hypothesis that planktonic forms derive from benthic ancestors (e.g., related to *Chaetonotus (Zonochaeta)* Remane, 1927; see Kisielewski 1991) has gained support from a recent phylogenetic study based on the analysis of molecular markers (Kånneby et al. 2013). This study has in fact found that the 11 Dasydytidae analysed (7 putative species) can be combined into a monophyletic group deeply nested within the Chaetonotidae as sister to a clade containing *Chaetonotus (Zonochaeta)* species. While shedding light on the probable origin of the planktonic forms, at least that of the Dasydytidae, the work by Kånneby et al. (2013) has also shown phylogenetic relationships that in part contrast with the current systematization of the group, e.g., *Dasydytes carvalloe* Kisielewski, 1991, appears basal to a clade composed of *Dasydytes* spp. and *Stylochaeta* spp. This highlights the fact that planktonic gastrotrichs are still too poorly known for reasonable confidence in the current phylogenetic inferences based on morphological traits to prevail. Uncertainties in this regard are also shown by the unstable systematics that plague some taxa (e.g., Kisielewski 1991 vs Schwank 1990 for Dasydytidae). This situation is due to the scarcity of information available, both at the level of the number of known species included in the currently recognized genera and subgenera (generally too low to provide reliable taxonomic boundaries) and of the techniques that have been utilized in the past to investigate these animals (in many instances inadequate to current standards). The Family Neogosseidae, and particularly the genus *Kijanebalola* Beauchamp, 1932, may be considered a paradigmatic example in both regards.

*Kijanebalola* includes the type species *Kijanebalola dubia* Beauchamp, 1932 from an Ugandan lake and *Kijanebalola canina* Kisielewski, 1991, from a pond in the State of Parà, Brazil. The status of a third species, described as a rotifer, *Eretmia cubeutes* Gosse in Hudson and Gosse 1886, is uncertain and will not be considered further. All species have been found only once. *Kijanebalola dubia* was established on the basis of two formalin-fixed, contracted specimens examined with light microscopy (very likely bright field) and, by admission of the author, the description is to be considered incomplete (Beauchamp 1932). Better is the situation for the second species, *Kijanebalola canina*, the description of which benefitted from the availability of numerous specimens (10). Most of these were relaxed with novocaine and examined in vivo using a bright field microscope, with some specimens fixed later and observed further with DIC optics (Kisielewski 1991). The thorough examination of the Brazilian species allowed the improvement of the diagnosis at familial and generic level (cf. Kisielewski 1991).

We report here on two interesting Neogosseidae found during an ongoing survey of the meiofauna and macrobenthos of the iSimangaliso Wetland Park (Pillay and Perissinotto 2009, Todaro et al. 2011, Bownes and Perissinotto 2012, Daly et al. 2012, Gómez et al. 2012). One species belongs to the genus *Neogossea* Remane, 1927 and is reported here for the first time outside of its Brazilian type locality, whereas the second appears to be new to science. The details of the anatomical characteristics of the latter, observed using a variety of techniques including electron microscopy, warrant a further revision of the diagnostic traits not only of the genus *Kijanebalola*, butactually of the entire Family Neogosseidae. Thus, beside increasing our knowledge of the biodiversity hosted by South Africa’s first UNESCO World Heritage Site (Taylor 2006), this study will be beneficial to future phylogenetic studies of Gastrotricha based on morphology, by allowing the selection and/or a more precise character coding of traits of phylogenetic relevance.

## Methods

**Figure 1.**
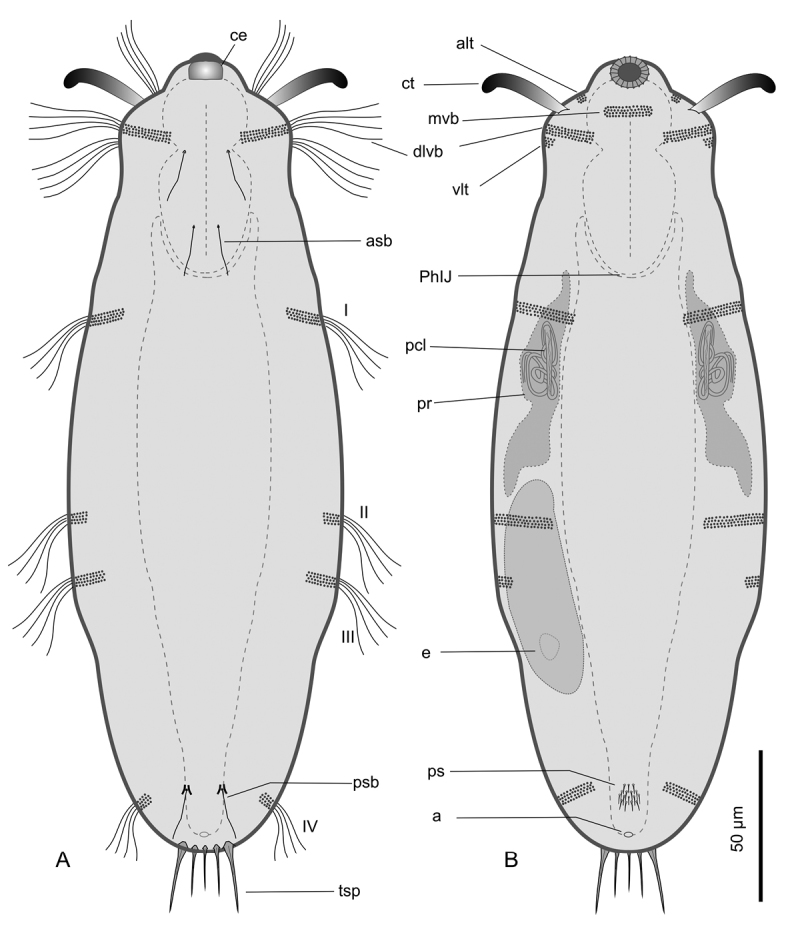
*Kijanebalola devestiva* sp. n. schematic drawings. **A** Dorsal habitus **B** Ventral habitus, showing some internal structures. **a** anus **alt** antero-lateral tuft of cephalic cilia **asb** anterior sensory bristle **ce** cephalion **ct** cephalic tentacle **dlvb** lateral band of cephalic cilia extending dorsally and ventrally **e** egg **I-IV,** first to fourth band of trunk ciliature **mvb** median ventral band of cephalic cilia **pcl** proximal canal cell lumen **PhIJ** pharyngeo-intestinal junction **ps** patch of keeled scales **psb** posterior sensory bristle **tsp** terminal spines **vlt** ventro-lateral band of cephalic cilia.

Samples containing gastrotrichs were collected on 11 February 2013 from a 310 m × 70 m freshwater pond located near Charter’s Creek on the Western Shores of Lake St Lucia, iSimangaliso Wetland Park, South Africa. The pond was unevenly divided in two interconnected-pools by a white road; its maximum depth was about 1-1.5 m and the water surface almost completely covered by aquatic vegetation, constituted mainly of blue waterlilies, *Nymphaea nouchali*. Collection was carried out in both pools by operating from the edge of the road with a hand-held plankton net (15 cm diameter, 25 μm mesh) used to scoop up water from the thick vegetation. The geographic coordinates of each site were recorded by means of a Garmin GPS-12 portable receiver, while the physico-chemical characteristics of the water were measured by means of a YSI 6600 multiprobe system. A list of the main characteristics of the sites surveyed is reported in Table 1 and a comprehensive map along with photos taken at the time of sampling are provided in the Appendix: Figure 1S.

**Table 1.** Physico-chemical data and geographic coordinates of the four sampling sites investigated at the pond near Charter’s Creek on the Western Shores of Lake St Lucia, iSimangaliso Wetland Park, South Africa. Pool A represents the larger pool situated on the right side of the road, travelling from Charter’s Creek towards St Lucia town. Pool B is the smaller pool on the left side of the same road (see also supplementary file Figure 1S).

**Variable**	**Pool A**		**Pool B**	
	**1**	**2**	**3**	**4**
Temperature (°C)	24.8	24.9	22.8	22.9
Conductivity (mS/cm)	0.24	0.25	0.25	0.40
Salinity	0.12	0.12	0.12	0.19
Dissolved O_2_ (%)	9.7	15.4	5.5	6.0
Dissolved O_2_ (mg/L)	0.78	1.23	0.46	0.5
pH	8.1	7.3	6.7	6.6
Turbidity (NTU)	21.5	17.6	16.8	14.3
Surface light intensity (µmol.cm^-2^.s^-1^)	184.9	222.4	242.6	179.1
Bottom light intensity (µmol.cm^-2^.s^-1^)	20.97	16.73	32.48	3.93
Depth (m)	0.5	0.7	0.60	0.80
Geographic coordinates	Latitude 28°15'19"S<br/> Longitude 32°23'37"E		Latitude 28°15'19"S<br/> Longitude 32°23'38"E	

A total of four 1 L plastic jars filled with water, some debris and little vegetation from each pool (2+2 stations) were brought back to the laboratory at the University of KwaZulu-Natal (Durban) within 24 hr and analysed during a one-week period. To extract gastrotrichs, samples were stirred with a plastic pipette and aliquots of the sediment-water mixture were poured into 10 cm diameter plastic Petri dishes and scanned under a Wild M5 stereo-microscope. The animals of interest were picked-out with a micro-pipette, transferred to a slide in a drop of 1% MgCl_2_ solution and studied live (Kånneby et al. 2009, Todaro et al. 2012). Photos and measurement were taken with a Nikon Eclipse 80i DIC microscope equipped with a Nikon Digital Sight DSFi1 digital camera and a Nikon Nis-Elements software. Some specimens were fixed in 10% borax-neutralized formalin and stored for later SEM analysis. To this end, gastrotrichs were rinsed in distilled water, dehydrated through a graded ethanol series, critical point-dried using CO_2_, mounted on aluminium stubs, sputter coated with gold-palladium and observed with a Philips XL 30 scanning electron microscope at the first author’s institution in Modena, Italy (cf. Todaro 2013). Five additional specimens were fixed and kept in absolute ethanol for future DNA analysis. The description of the new species follows the convention of Hummon et al. (1992), whereas the position of key morphological characters is given in percentage units (U) of total body length, measured from anterior to posterior end, excluding the terminal spines.

Abbreviations used in the text are as follows: **CT** cephalic tentacles; **PhIJ**, pharyngeo-intestinal junction; **PhL**, pharynx length, **TL**, total length.

## Taxonomic account

### Order Chaetonotida Remane, 1925 [Rao & Clausen, 1970]
Suborder Paucitubulatina d’Hondt, 1971
Family Neogosseidae Remane, 1927
Genus *Kijanebalola* Beauchamp, 1932

#### Kijanebalola devestiva sp. n.

urn:lsid:zoobank.org:act:53BA8B47-3BC3-4AD1-9B5F-1CBD5F9F34DB

http://species-id.net/wiki/Kijanebalola_devestiva

Figs 1 [Fig F2] [Fig F3] [Fig F4] [Fig F5] –6

##### Type locality.

Roadside freshwater pond near Charter’s Creek, Lake St Lucia, Western Shores, iSimangaliso Wetland Park, South Africa (Lat. 28°15'19"S; Long. 32°23'37"E; Table 1, Figure 1S).

##### Type specimens.

**Figure 2. F2:**
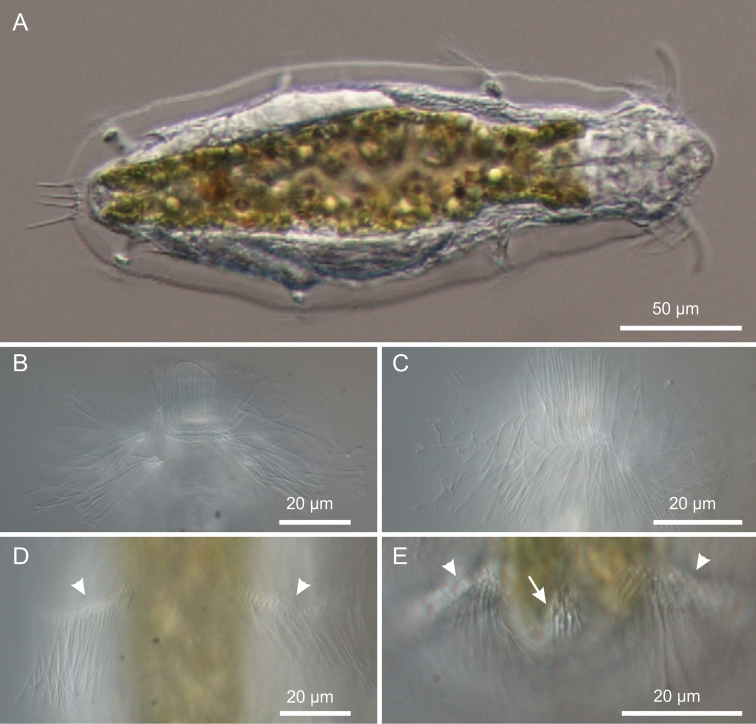
*Kijanebalola devestiva* sp. n. DIC photomicrographs. **A** habitus **B**, **C** close-up ventral view of theanterior region showing the ciliary bands **D** close-up ventral view of the mid-trunk region showing the second ciliary bands **E** close-up ventral view, of the posterior region, showing the fourth ciliary bands (arrowheads) and the residual patch of spined scales (arrow).

Holotype: adult specimen 267 μm long shown in Figure 2, no longer extant (International Code of Zoological Nomenclature, Articles 73.1.1 and 73.1.4).

##### Material examined.

Thirteenspecimens (including the holotype) collected by the first author (10 from pond A and 3 from pond B, see Table 1). Seven specimens were observed alive and are not longer extant, while six were prepared for SEM analysis and are kept in the meiofauna collection of the first author (Ref. n. 2013-SA-01-02).

##### Ecology.

Above silty substratum, among vegetation.

##### Diagnosis.

A *Kijanebalola* with an adult length to 310 μm; body roughly barrel-shaped with head weakly separated from trunk and the rounded-off posterior end exhibiting medially a group of five spines; head with a pair of 26 μm long, club-like tentacles and a shallow cephalion; cuticular covering mostly smooth, except for a tiny patch of small triangular, keeled scales on the ventral side at the rear trunk; locomotor cilia arranged in tufts and interrupted bands on the head and 4 paired transverse bands along the trunk; three pairs of 14–16 μm long sensory bristles on the dorsal side, at U12, U22 and U92; mouth 13 μm in diameter, slightly protruding down forward and reinforced internally by 17-20 thick, longitudinal ridges; pharynx up to 67 μm, consisting of anterior spherical and posterior nosecone-shaped bulbs; PhIJ at U27; intestine straight, with anterior portion embracing the posterior portion of the pharynx; one pair of conspicuous protonephridia located adjacent to the intestine, from the PhIJ to about mid-body; parthenogenetic.

##### Etymology.

The specific name *devestiva* (from the Latin, *devestivus*, undressed), alludes to the general absence of cuticular ornamentations, such as scales and spines that cover the body of other congeneric species.

##### Description.

This description is mainly based on an adult specimen, 267 μm in total length (TL, posterior spines excluded). The body is roughly barrel-shaped with the head weakly separated from the trunk by a slight neck constriction and the posterior trunk region rounded-off, without paired lateral projections but exhibiting medially a group of five spines. Body widths at the head/neck/trunk/caudum are 57/55/90/32 μm, at U09/14/52/97, respectively. The head is provided with a pair of club-like tentacles projecting antero-laterally; they are 26 μm in length and insert ventro-laterally at U07; the hypostomion is absent; a shallow cephalion (10 × 4.5 μm) is appreciable only under SEM (Figure 5C). Under dissecting microscope, the animals appear swimming slowly in a rectilinear direction, with some following loose helicoidal trajectories. When purposely stimulated with a needle, specimens react by escaping aside, but never retracting the head inside the body; by contrast, most of the fixed specimens appear to have the head retracted to some extent (Figure 6A).

*Cuticular armour*. The body is covered by a smooth cuticle, except for a minute patch of keeled scales located on the ventral side of the posterior trunk region, at U93 (Figures 1B, 2E). The scales, arranged in 5–7 columns of 3–5 scales each, are very small (ca 1 μm) and may go undetected under light microscopy (DIC); when observed with a scanning electron microscope, they appear roughly triangular in shape and their keel continues in a proportionally long spiny process (Figure 6C). Five robust terminal spines, 15–24 μm in length, ornate the posterior end of the trunk; they are inserted dorsally to the anus (Figure 6B, D).

*Ciliation*. Locomotory cilia are arranged in tufts and interrupted bands around the head and paired transverse bands along the trunk (Figures 1, 2A–E, 5A, B). Most of the cephalic cilia have a ventral or ventro-lateral distribution; however, a precise organization is difficult to see due to their high density and relatively long span (16-18 μm in length). From anterior to posterior end, it is possible to discern the following groups: a pair of antero-lateral tufts, a median ventral band, a pair of lateral bands extending ventrally and dorsally, followed by a pair of ventro-lateral tufts (Figure 1). The trunk ciliature consists of four pairs of oblique short bands, with first (U32) and fourth (U94) inserted dorso-latero-ventrally, the second (U59) inserted latero-ventrally and the third (U66) latero-dorsally (Figures 1, 2D, E, 5A).

Three pairs of sensory bristles (14–16 μm in length) are present on the dorsal side at U12, U22 and U92, respectively (Figures 1A, 4B, C). The bristles of the first two pairs emerge from round pits, while posterior bristles originate directly from the cuticle and are flanked by two anteriorly-converging keels. Presence of additional sensory bristles hidden among the cephalic locomotor ciliation cannot be excluded.

**Figure 3. F3:**
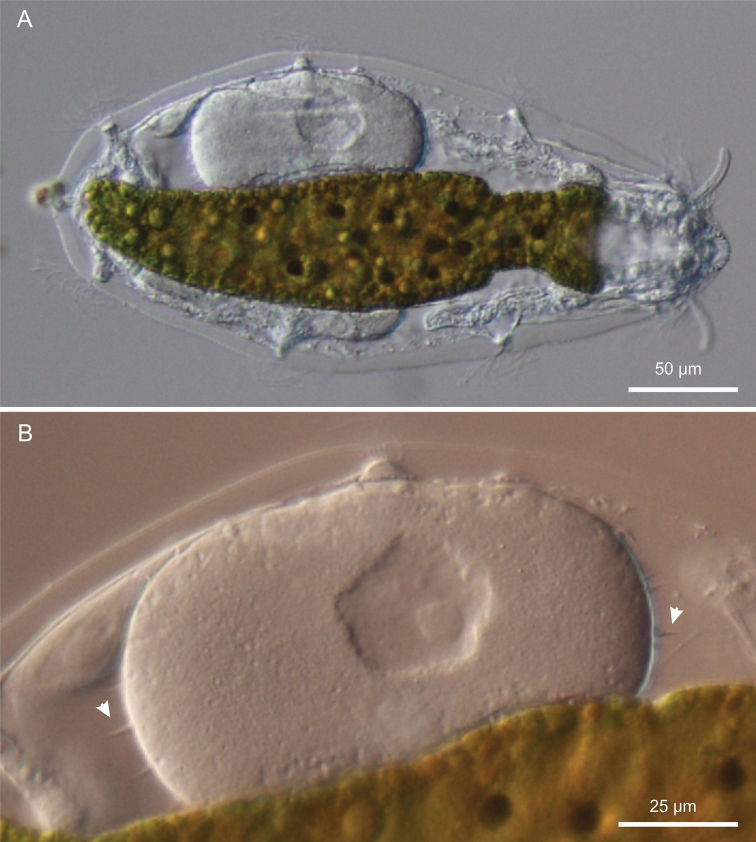
*Kijanebalola devestiva* sp. n. DIC photomicrographs. **A** habitus of a gravid specimen **B** close-up view of the inside egg with the shell bearing spine-like ornamentation (arrowheads).

**Figure 4. F4:**
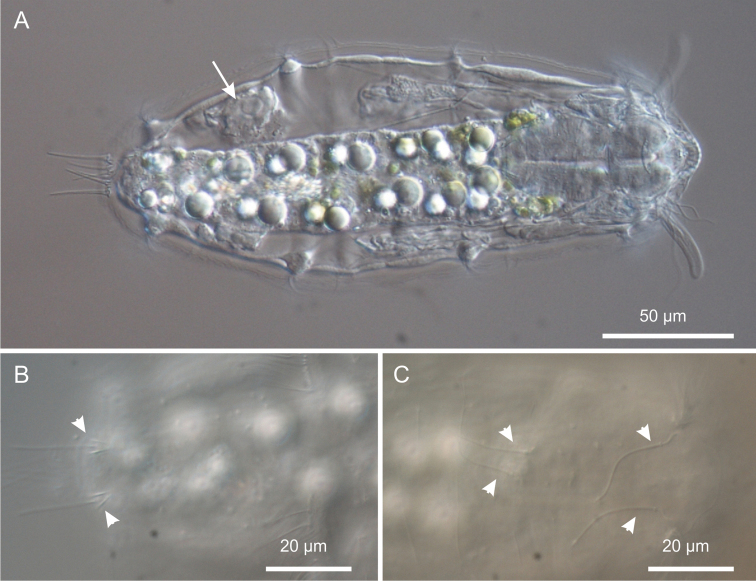
*Kijanebalola devestiva* sp. n. DIC photomicrographs. **A** habitus of a subadult specimen showing developing egg (arrow) **B** close-up dorsal view of the posterior trunk region showing the two sensory bristles (arrow-heads) **C** close-up dorsal view of the anterior trunk and neck regions, showing two pairs of sensory bristles.

**Figure 5. F5:**
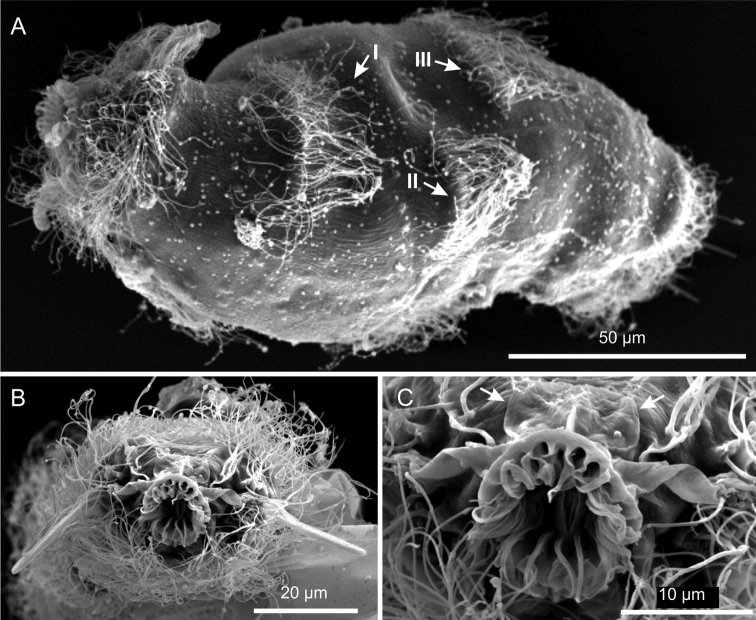
*Kijanebalola devestiva* sp. n. SEM photomicrographs. **A** habitus in a ventro-lateral view; note the arrangement of the first, second and third ciliary bands on the trunk region (number and arrows) **B** anterior region of a different specimen in a frontal view **C** close-up view of the mouth ring and cephalion (arrows).

**Figure 6.**
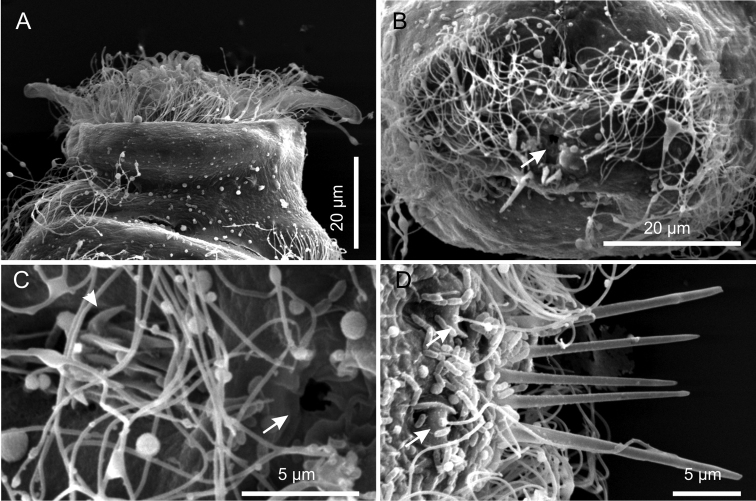
*Kijanebalola devestiva* sp. n. SEM photomicrographs. **A** anterior region of specimen with head partially retracted inside the body (ventral view) **B** posterior trunk region of different specimen, showing the fourth ciliary band and the anus (arrow) **C** close-up view of the posterior end showing the anus (arrow) and the residual patch of spined scales (arrows) **D** close-up view of the posterior end (dorsal view), showing theterminal spines and the sensorial bristles (arrows).

*Digestive tract*: The strong mouth ring is terminal and about 13 μm in diameter; it appears slightly protruding down forward and is reinforced inside by 17–20 thick longitudinal cuticular ridges, which protrude externally and bend on the outer contour (Figure 5). The pharynx is 64 μm in length and shows anterior and posterior bulbs separated by a noticeable constriction; the anterior bulb, deprived of cuticular reinforcement, is roughly spherical (28 × 24 μm), while the posterior one (30 × 40 μm) is more nosecone-shaped (Figures 1, 4A); PhIJ is at U27. The intestine is straight; in the adult it appears impressively filled with green material (Figures 2A, 3A) while in juveniles it is packed with translucent globules (Figure 4A). Peculiarly, the anterior portion of the intestine extends forward encircling a large part of the posterior portion of the pharynx (about half the length of the posterior bulb), resulting in this region much wider than the pharynx itself (36 μm); at the PhIJ and for a short tract the intestine is about as wide as the pharynx (28–30 μm), then it widens again reaching a maximum width of 45 μm at about mid-body (U52); after this point the gut progressively narrows until it joins the 5–6 μm sub-terminal anus at U97 (Figures 1, 6B, C).

*Nephridial system*. There is a pair of conspicuous protonephridia adjacent to the intestine; each protonephridium occupies an area extending from the PhIJ to about mid-body (U27-U53) and includes a clearly visible tubular canal containing two vibrating flagella, corresponding to the proximal canal cell lumen of Kieneke et al. (2008a). This canal is about 25 μm in length and runs almost parallel to the intestine, slightly converging towards the gut with its posterior portion (see Appendix: Figure 2S).

*Reproductive tract*. All the adult specimens were in parthenogenetic phase, with respective eggs at different stages of development.

*Variability and remarks*. The largest adult was about 310 μm in total length (terminal spines excluded) and was carrying a very big egg inside (73 × 107 μm, Figure 3A). Remarkably, the egg shell was ornated with spikes (Figure 3B); this is quite surprising because in freshwater Gastrotricha the shell ornamentation is believed to appear only after the egg has been laid, probably due to osmotic differences between the external vs internal milieu (Hummon 1984). The smallest animal was about 183 μm in total length and had no recognizable female gametes inside. Oocytes became appreciable as such in a 230 μm long specimen (Figure 4A).

##### Taxonomic affinities.

The general body appearance, the presence of club-shaped cephalic tentacles and the planktonic lifestyle that characterise *Kijanebalola devestiva* sp. n. suggest that the closest affiliation of the South African specimens lies with the Neogosseidae. Notwithstanding this, autoapomorphic traits of the new species make somewhat difficult its affiliation to any of the two currently recognized genera, *Neogossea* and *Kijanebalola*, based on their current diagnosis (see Kisielewski 1991). For instance, the size of the South African worms exceed by far that of any other possible keen (max length 310 μm vs 200 μm in *Neogossea* spp., vs 210 in *Kijanebalola* spp.) and the body covering, made for most part of smooth cuticle, is a characteristic so far unknown among species of *Neogossea* or *Kijanebalola*. The presence in the new species of a single cephalic plate (i.e., the cephalion), combined with the structure of its mouth and pharynx, complicate further its taxonomic affiliation at generic level. However, in our opinion, some of the differences between the anatomical traits of the iSimangaliso gastrotrichs and those highlighted in the diagnosis of *Neogossea* or *Kijanebalola* no longer hold. Regarding *Neogossea*, for instance, the diagnosis: 1) is ambiguous about the presence of cephalic plates (i.e., cephalion and hypostomion reported as undetected); and 2) includes the mouth as consisting of two-segmented units (mouth units = internal cuticular ridges). A re-evaluation of these characteristics, especially in light of the new information gained by Kieneke and Riemann (2007) on German specimens of *Neogossea* (reported as *Neogossea voigti* (Daday, 1905) allows for the amendment of the generic diagnosis of *Neogossea* in both these aspects. More specifically, the specimens studied by Kieneke and Riemann (2007) under differential interference contrast and scanning electron microscopy show: 1) the hypostomion and 2) the mouth units as unsegmented. Considering that in Neogosseidae the presence of a cephalion may be elusive (as reported above for the new species) we conclude that the presence/absence of the cephalic plates and the structure of the mouth and pharynx cannot be considered diagnostic characters at genus level (i.e., useful to distinguishing *Neogossea* from *Kijanebala* and *vice versa*); rather, peculiarities in these traits and others such as the presence/absence of cuticular reinforcements in the anterior pharyngeal bulb, may help differentiate among species (i.e., they are species specific).

Another potential difference between the two genera i.e., the presumed ability of *Kijanebalola* species to partially retract the head inside the body, can also be dismissed. Our observations with the new species demonstrate that retraction of the head is actually an artefact due to fixation (see above). Consequently, none of the above reported traits may be used to allocate our specimens to either of the two genera.

In summary, only a single character is apparently left to differentiate *Neogossea* and *Kijanebalola*; it pertains to the structure of the posterior part of the trunk, which appears truncate with a pair of postero-lateral projections, each provided with a tuft of relatively long spines in *Neogossea*, but rounded-off with a median group of spines in *Kijanebalola*. As the difference in this character between species of the two genera is consistent, it is reasonable to use it as diagnostic feature and as a solid synapomorphy for each genus.

Consequently, based on the round shape of the posterior trunk region of the iSimangaliso specimens, we propose that their closest affiliation is to the genus *Kijanebalola*. Therefore, the large size, the cuticular ornamentation reduced to an epaulet of scales on the ventral side of the trunk ending, and the number and size of the terminal spines are considered autoapomorphic characters that can easily distinguish *Kijanebalola devestiva* sp. n. from *Kijanebalola dubia* and *Kijanebalola canina*. Emended diagnoses are reported below.

### Genus *Neogossea* (Remane, 1927)

#### Neogossea acanthocolla

Kisielewski, 1991

http://species-id.net/wiki/Neogossea_acanthocolla

Fig. 7

##### Material.

2 adult specimens (1 measured and documented), South Africa, KwaZulu-Natal, roadside freshwater pond near Charter’s Creek, Lake St Lucia, Western Shores, 11 February 2013, MA Todaro legit.

##### Morphometry.

TL, 122 μm (posterior spines excluded); PhL, 34.5 μm; PhIJ at U36; CT, 24.5 μm; dorsal cuticular covering made up of about 16 columns of 17–24 trilobed scales bearing a short, simple spine; a group of 18 densely packed spines is present on the dorsal side of the neck; spines are 10–13 μm in length, rather thick and with a notched tip; posterior tufts of long spines made up of 7 spines each; spines are 45–50 μm in length and barbed, with the lateral denticle positioned at about 3/4 of the spine length.

**Figure 7.**
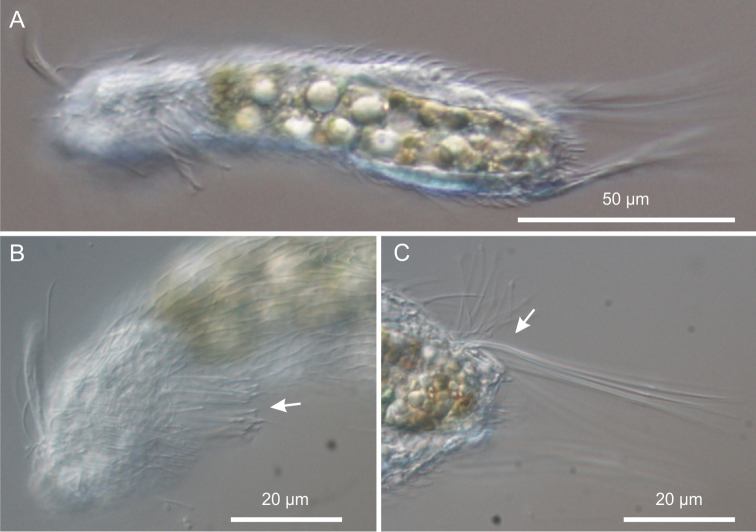
*Neogossea acanthocolla*. DIC photomicrographs. **A** habitus **B** anterior region showing the group of thick spines on the neck (arrow) **C** close-up of the posterior region of the trunk showing a tuft of long, barbed spines (arrow).

##### Remarks.

Morphometry and general appearance of the specimen from the iSimangaliso Wetland Park are in general accordance with data reported for the Brazilian *Neogossea acanthocolla*, the main peculiarity of which is the presence of the brush-like group of spines on the neck. A noticeable difference between the South American and African specimens lies in the type of scales covering the body: pedunculated and rhomboidal in the former, but ordinary (trilobed) and spined in the latter. However, as similar differences have been reported among members of the Brazilian populations (cf. Kisielewski 1991), it may be cautious not to regard this trait alone as a discriminatory character. Future studies, accounting for morphological and/or molecular marker differences on a statistical basis could support or disprove this hypothesis (e.g., Todaro et al. 1996, Leasi and Todaro 2009, Kånneby et al. 2012, Kieneke et al. 2012).

## Diagnoses

### Family Neogosseidae Remane, 1927 (emended diagnosis)

Paucitubulatina exhibiting weakly flattened body, 90–310 μm in length (posterior spines excluded). With a pair of club-shaped cephalic tentacles. Caudal furca and adhesive tubes absent. Posterior trunk with paired postero-lateral projection or rounded-off. Locomotor ciliature consisting of tufts and interrupted bands around the head and several pairs of tufts or short obliquely-running bands along the trunk. Cephalion and hypostomion present or absent. Body covered with scales, rarely naked with scales reduced to an epaulet on the ventral side; scales in general spined rarely pedunculated; spines simple, occasionally barbed. Trunk end with long spines distributed in paired lateral and/or unpaired median group. Mouth ring large and terminal, reinforced inside by thick longitudinal cuticular ridges, which may protrude externally. Pharynx strong with one to four bulbs. With a pair of protonephridia in the anterior trunk region. Parthenogenetic, with paired ovary. Male system unknown. Freshwater, semipelagic and/or planktonic.

### Genus *Neogossea* Remane, 1927 (emended diagnosis)

Neogosseidae with body 90–200 μm in length (caudal spines excluded). Posterior end of body truncate, with a pair of postero-lateral projections, each provided with a tuft of long spines, simple or barbed, occasionally also with short claw-like structure. Cephalion and hypostomion present or absent. Body covered with fine and spined scales; scales trilobed or with edges often fused with basal cuticle; occasionally and only partially with pedunculated scales. Spines short and simple, occasionally partly long and barbed. Mouth ring large and terminal, reinforced inside by thick longitudinal cuticular ridges, protruding externally. Anterior half of pharynx consisting of terminal bulb followed by two smaller dilations; posterior half in the form of large bulb. Cuticular pharyngeal reinforcements lacking. Six species: *Neogossea antennigera* (Gosse, 1851) - type-species, *Neogossea acanthocolla* Kisielewski, 1991, *Neogossea fasciculata* (Daday, 1905), *Neogossea pauciseta* (Daday, 1905), *Neogossea voigti* (Daday, 1905) and *Neogossea sexiseta* Krivanek & Krivanek, 1959.

### Genus *Kijanebalola* Beauchamp, 1932 (emended diagnosis)

Neogosseidae exhibiting body 135–310 μm in length (caudal spines excluded). Posterior end of trunk rounded-off, with median group of spines and without lateral protrusions. Cephalion and hypostomion present. Body covered with convex scales with median keels and rudimentary spines, the latter tend to prolong in some trunk portions; occasionally body mostly naked with scales reduced to an epaulet on the ventral side. Pharynx consisting of at most two bulbs. Anterior pharynx portion at least as thick as posterior one and occasionally provided with strong and complex system of cuticular reinforcements. Mouth ring large and terminal, reinforced inside by thick longitudinal cuticular ridges, which may protrude externally.

Three species: *Kijanebalola dubia* Beauchamp, 1932 (type-species), *Kijanebalola canina* Kisielewksi, 1991 and *Kijanebalola devestiva* sp. n. The status of an additional species, originally described as a rotifer, i.e., *Eretmia cubeutes* Gosse in Hudson and Gosse 1886, but potentially belonging to the genus *Kijanebalola* as fourth species (see Beauchamp 1932 and Remane 1936) should be adequately re-assessed before a formal decision is made in this regard.

## Supplementary Material

XML Treatment for Kijanebalola devestiva

XML Treatment for Neogossea acanthocolla

## Appendix

Comprehensive map and DIC photomicrograph. (doi: 10.3897/zookeys.315.5593.app) File format: JPEG Image (jpg). **Explanation note:** Figure 1S. Sampling location. Comprehensive map along with photos taken at the time of sampling. Figure 2S. *Kijanebalola devestiva* sp. n. DIC photomicrograph. Close-up of the protonephridial apparatus.
